# Band Structure and Energy Level Alignment of Chiral Graphene Nanoribbons on Silver Surfaces

**DOI:** 10.3390/nano11123303

**Published:** 2021-12-06

**Authors:** Martina Corso, Rodrigo E. Menchón, Ignacio Piquero-Zulaica, Manuel Vilas-Varela, J. Enrique Ortega, Diego Peña, Aran Garcia-Lekue, Dimas G. de Oteyza

**Affiliations:** 1 Centro de Física de Materiales (MPC), CSIC-UPV/EHU, 20018 San Sebastián, Spain; ge46biq@mytum.de (I.P.-Z.); enrique.ortega@ehu.es (J.E.O.); 2 Donostia International Physics Center (DIPC), 20018 San Sebastián, Spain; menchon@dipc.org; 3 Department de Polímeros y Materiales Avanzados: Física, Química y Tecnología, Universidad del País Vasco (UPV/EHU), 20080 San Sebastián, Spain; diego.pena@usc.es; 4 Centro Singular de Investigación en Química Biolóxica e Materiais Moleculares (CiQUS), Departamento de Química Orgánica, Universidade de Santiago de Compostela, 15782 Santiago de Compostela, Spain; manuel.vilas.varela@usc.es; 5 Department Física Aplicada I, Universidad del País Vasco, 20018 San Sebastián, Spain; 6 Ikerbasque, Basque Foundation for Science, 48013 Bilbao, Spain

**Keywords:** graphene nanoribbons, edge states, interface energetics, charge transfer, spin polarization

## Abstract

Chiral graphene nanoribbons are extremely interesting structures due to their narrow band gaps and potential development of spin-polarized edge states. Here, we study their band structure on low work function silver surfaces. The use of a curved Ag single crystal provides, within the same sample, regions of disparate step structure and step density. Whereas the former leads to distinct azimuthal growth orientations of the graphene nanoribbons atop, the latter modulates the substrate’s work function and thereby the interface energy level alignment. In turn, we disclose the associated charge transfer from the substrate to the ribbon and assess its effect on the nanoribbon’s properties and the edge state magnetization.

## 1. Introduction

Carbon-based nanostructures can display exceptionally varied properties depending on their precise bonding structure. This includes graphene nanoribbons (GNRs) [1,2,3], in which a graphene lattice is confined to narrow, one-dimensional stripes. GNRs with armchair-oriented edges display a semiconducting band structure. In contrast, zigzag and even chiral GNRs are quasi-metallic and develop spin-polarized edge states [2,3,4,5], unless they are exceedingly narrow. In this case, the edge states from either side hybridize with one another, which quenches the spin polarization and confers the ribbons a conventional semiconducting band structure [6,7].

For ribbons with a (3,1) chiral vector, the minimum width required to maintain the quasi-metallic behavior comprises six carbon zigzag lines from side to side [6]. This theoretical prediction has been recently confirmed experimentally by synthesizing and spectroscopically characterizing (3,1) chiral GNRs of varying widths on Au(111) [8]. However, these ribbons, as well as purely zigzag edged GNRs [9] or other GNRs featuring low energy states associated with periodic zigzag edge segments [10,11,12], have been synthesized and characterized to date only on Au(111).

To investigate the effect of different substrates with a lower work function on the ribbon’s electronic properties, here we synthesize six zigzag lines wide (3,1) chiral GNRs ((3,1,6)-chGNRs) on a curved Ag crystal [13] that spans up to ±15 degrees of vicinal angle α to either side with respect to the central (111) surface orientation. The synthesis is successful over the entire crystal, but the different types of steps on each side of the sample have a disparate effect on the ribbon’s preferred azimuthal alignment. This provides us with an ideal sample on which to study the band dispersion by angle-resolved photoemission (ARPES), both along and perpendicular to the ribbon’s longitudinal axis.

## 2. Materials and Methods

**Figure 1:**
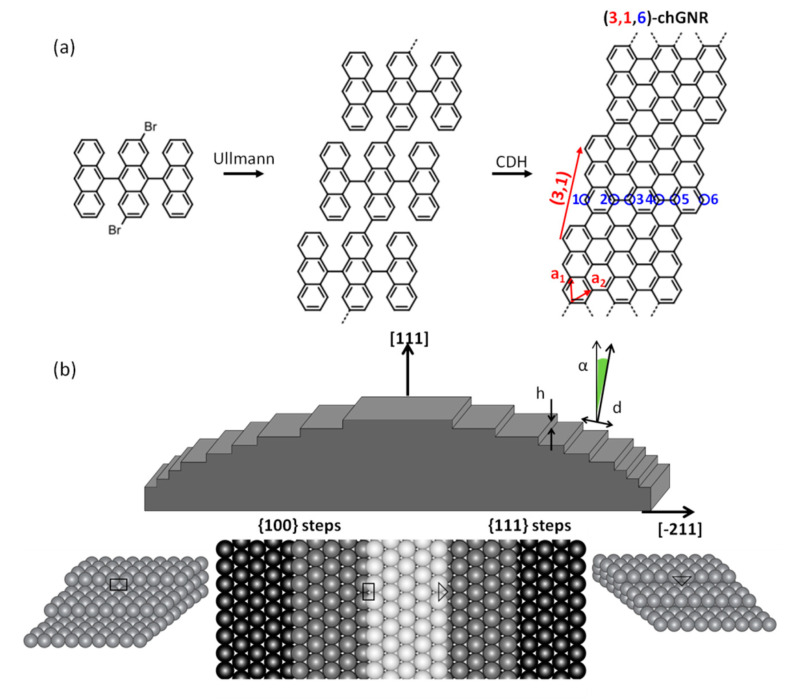
(**a**) Reactant (DBTA) and reaction scheme towards the (3,1,6)-chGNR structure, displaying a (3,1) chiral vector marked in red and six atoms across its width marked in blue. (**b**) Schematic description of the Ag curved crystal where *d* corresponds to the terrace width, *α* to the vicinal angle from the [111] direction, and *h* to the monoatomic step height. The steps at the left and right sides of the crystal display {100}-oriented and {111}-oriented microfactes, respectively.

The reactant molecules 2′,6′-dibromo-9,9′:10′,9′′-teranthracene (DBTA, Figure 1a) were synthesized as reported in an earlier study [8]. The employed curved silver crystal was prepared with standard sputtering/annealing parameters (E = 1000 eV/T = 370 °C). The reactant molecules were sublimed from a homemade Knudsen cell heated to a temperature of around 265 °C at a rate of 0.06 ML/min as controlled with a calibrated quartz crystal microbalance. The sample was subsequently annealed to 180 °C and 315 °C for 10 min and 1 min, respectively, to separately activate the polymerization and cyclodehydrogenation steps. The sample was first analyzed with STM and subsequently transferred to the ARPES chamber without breaking the vacuum. The STM images were acquired at room temperature with a commercial Omicron VT-STM and processed with the WSXM software [14]. ARPES measurements were obtained with a high-intensity monochromatic source (21.2 eV) and a high-resolution display-type hemispherical electron analyzer (Phoibos150). The vertically aligned manipulator and analyzer slit were perpendicular to the horizontally aligned step direction of the curved crystal, allowing measurements over a wide band dispersion range parallel to the steps by sample rotation (polar scans by manipulator rotation). The sample temperature during the ARPES experiments was approximately 150 K. The lab-based He gas discharge lamp has a spot size of approximately 1 mm^2^, therefore by moving the sample perpendicular to the steps in steps of 1 mm, it is possible to probe sample regions with different step densities (as shown in Supplementary Figures S1 and S2).

First-principles electronic structure calculations were performed using the DFT as implemented in the SIESTA software package [15,16]. The van der Waals density functional by Dion et al. [17] with the modified exchange correlation by Klimeš, Bowler, and Michaelides [18] was used. The valence electrons were described by a double-ζ plus a polarization (DZP) basis set with the orbital radii defined using a 54 meV energy shift [16], while the core electrons were described using norm-conserving Trouillers-Martins pseudopotentials [19]. For integrations in real space [16], an energy cutoff of 300 Ry was used. The smearing of the electronic occupations was defined by an electronic temperature of 300 K with a Fermi-Dirac distribution. The selfconsistency cycles were stopped when variations on the elements of the density matrix were less than 10^−4^ eV and less than 10^−4^ eV for the Hamiltonian matrix elements. In order to avoid interactions with periodic images from neighboring cells, systems were calculated within a simulation cell where at least 50 Å of the vacuum space was considered. Variable cell relaxations and geometry optimizations were performed using the conjugate gradient method using a force tolerance equal to 10 meV/Å and 0.2 GPa as a stress tolerance. A 101 k-point mesh along the GNRs’ periodic direction was used. In order to simulate charged nanoribbons with large portions of vacuum, the net charge of the systems was set to be different from zero while simultaneously adding a compensating background charge.

## 3. Results

The reactant DBTA transforms into (3,1,6)-chGNRs following a two-step process that consists of thermally activated Ullmann coupling and cyclodehydrogenation (CDH, Figure 1a) [8]. The substrate is a Ag single crystal curved around the [1,1,1] axis, with the (111) surface plane at the crystal’s central area as displayed in Figure 1b [13]. The stepped surfaces towards either side thus share the same (111) terrace structure (of varying width *d* depending on the vicinal angle). However, the steps display nonequivalent facets, namely, {100} facets on the left-hand side and {111} facets on the right-hand side (Figure 1b) [13].

**Figure 2:**
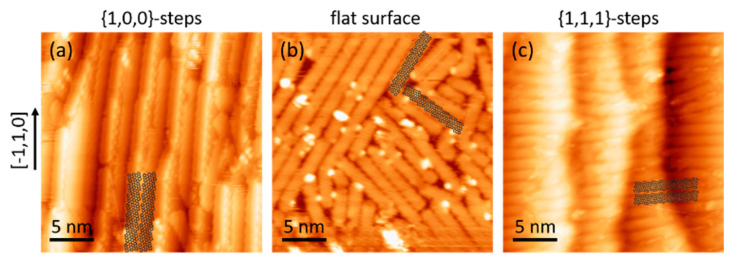
Representative STM images of the sample after GNR synthesis in regions with {100}-steps (**a**), on the flat (111) surface in the central crystal region (**b**), and with {111}-steps (**c**). The [−1,1,0] direction that coincides with the steps’ direction is shown on the left. Segments of two GNR structures are superimposed on each of the images as a guide to the eye.

Figure 2 shows representative images of the sample after depositing nearly a full monolayer of precursor molecules (DBTA) and stepwise annealing the crystal to 180 °C for 10 min and to 315 °C for 1 min, to drive the subsequent activation of polymerization and cyclodehydrogenation. Notably, the resulting GNRs display three distinct arrangements depending on the region of the Ag crystal.

In the stepped regions characterized by {100} facets, the GNRs are found preferentially aligned parallel to the steps’ direction (Figure 2a). In fact, in addition to the uniaxially aligned ribbons on top of the flat terraces, the ribbons display a particular affinity to the steps, adsorbing in a tilted configuration with either side of the GNR on each of the two neighboring terraces (Figure 2a). Both of these findings were expected, since stepped surfaces have been used often for alignment purposes [7,20,21,22,23,24], and many molecules, including GNRs [22,25], are known to display a particular affinity for adsorption on the undercoordinated and thus more reactive step atoms. Around the central (111) substrate orientation, displaying ample flat terraces, the ribbons adsorb with multiple azimuthal orientations, as expected from the six-fold symmetry of the surface (Figure 2b). In the stepped regions characterized by {111} facets, the GNRs display again uniaxially aligned ribbons. However, the ribbons surprisingly align perpendicularly rather than parallelly to the steps’ direction and extend over multiple terraces (Figure 2c). Taking into account that the terraces on the right and left-hand side of the crystal are identical, the difference in the preferential alignment must necessarily have its origin in the nature of the steps, which are formed by {100} and {111} facets, respectively. The specific interactions that cause this striking difference are beyond the scope of this work, but the resulting sample is ideal to probe the band dispersion parallel and perpendicular to the ribbon’s axis by ARPES.

**Figure 3:**
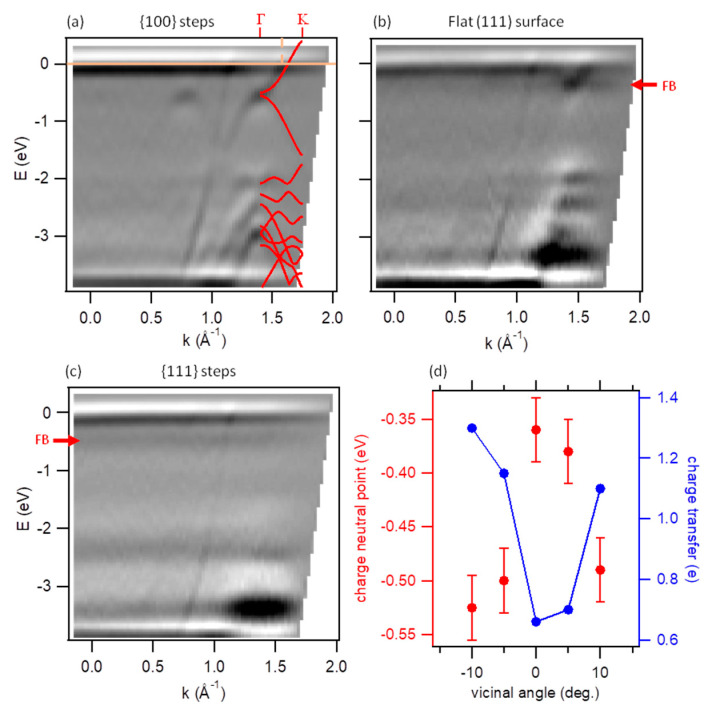
ARPES data displaying the dispersion along the [−1,1,0] direction of the curved Ag crystal on the stepped regions with {100} step facets (**a**, α ≈ −10°), on the central flat region (**b**, α ≈ 0°), and on the stepped region with {111} step facets (**c**, α ≈ 10°). The calculated band structure for free-standing GNRs after shifting the charge neutral point to −0.52 eV is superimposed on the third Brillouin zone of the panel (**a**). The horizontal light blue solid line marks the Fermi energy, and the vertical light blue dashed line marks its crossing point with the CB. The red arrows in panels (**b**) and (**c**) mark the flat band (FB) at the charge neutral point. (**d**) Measured CNP as a function of the vicinal angle and calculated charge transfer to reach such interface band alignment, according to the DFT.

We performed ARPES measurements that recorded the dispersion parallel to the [−1,1,0] substrate direction, which coincides with the step direction on both sides of the crystal. For the GNR bands, it corresponds to the dispersion along the longitudinal (Figure 3a) and transverse direction of the ribbons (Figure 3c) in the regions with {100} and {111} facets, respectively. The raw data along with the reference measurements on the clean crystal are displayed in Figure S1. As previously observed with narrower (3,1,4)-chGNRs [7], the band dispersion along the longitudinal ribbon direction (Figure 3a) is hardly recognizable in the first Brillouin zone, starts becoming visible in the second, and appears most intense in the third Brillouin zone (centred around 1.4 Å^−1^). Indeed, in the third Brillouin zone, not only the valence band but also following bands are observed with remarkable clarity, allowing for a direct comparison with the band structure predicted by DFT calculations for free-standing ribbons. As pictured in Figure 3a with the calculated bands superimposed on the ARPES data, there is an excellent match between experiment and theory. Such a good match, however, requires shifting the charge neutrality point (CNP) by −0.52 eV.

This shift implies a charge transfer at the GNR/silver interface. In contrast to Au, on which the GNRs show a clear tendency to become *p*-doped [8,26], the substantially lower work function of silver (e.g., 4.6 eV as compared to 5.4 eV for the (111) surfaces of Ag and Au, [27] respectively) favors the opposite electron transfer from surface to GNR. For the ARPES characterization, whereby only filled states are accessed, this has the advantage that also the conduction band can be probed. The extent to which the conduction band becomes accessible (populated) is quantitatively related to the charge transfer, taking into consideration that each band hosts two electrons per unit cell. The measurements in Figure 3a display 51% of the conduction band below the Fermi level, from where we can conclude that approximately one electron per GNR unit cell is transferred from the silver surface to the (3,1,6)-chGNRs, in qualitative agreement with the 1.3 electrons required to shift the CNP by 0.52 eV according to DFT calculations.

However, the amount of charge transfer shows variations across the curved silver surface. Figure 3c displays the dispersion along the transverse ribbon’s axis. Along this direction, the electronic states do not show any notable dispersion and appear as flat bands. This implies a negligible overlap of the wave functions of electronic states in neighboring ribbons. The extent to which the conduction band is populated cannot be inferred from these data as clearly as before. Yet, the flat band associated with the charge neutrality point appears at a similar energy as in Figure 3a, and hence the charge transfer can be concluded to be comparable.

The situation is slightly different in the central (111) region of the crystal (Figure 3b). There, the ribbons display multiple azimuthal orientations, each of them contributing to the convoluted ARPES signal. The overall dispersion can thus be recognized less clearly, although the general appearance can be ascribed to a washed-out convolution of Figure 3a,c. However, the CNP appears about 0.16 eV higher in energy. As a result, only 31% of the conduction band is populated, which in turn implies a charge transfer of only about 2/3 of an electron per unit cell. The modulation of the CNP as a function of the vicinal angle is displayed in Figure 3d (see the associated data in Figure S2) and is ascribed to the lower work function in the stepped regions as compared to the compact flat surface [7,22,28]. Figure 3d also displays the charge transfer required to shift the CNP to the measured energies, according to DFT calculations, and underlines the importance of local work function variations for influencing the electronic properties in weakly interacting metal-organic interfaces [29,30].

## 4. Discussion

**Figure 4:**
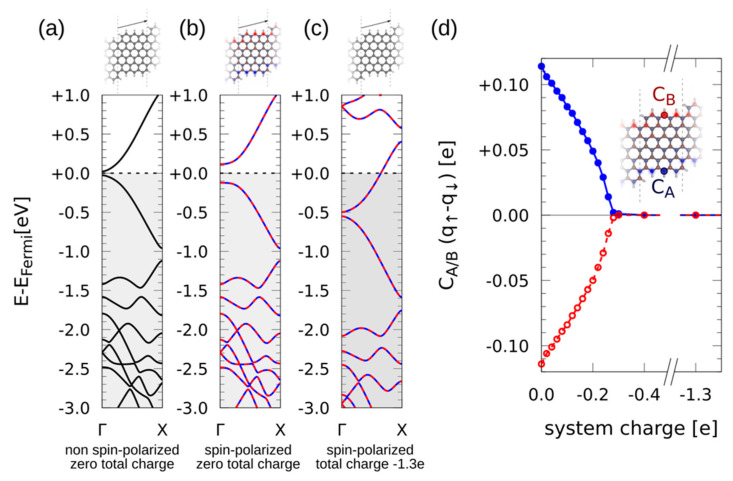
DFT simulations for (3,1,6)-chGNRs. (**a**) Relaxed atomic configuration (top) and electronic band structure (bottom) without spin-polarization. (**b**,**c**) Spin density (top) and spin-polarized band structure (bottom) for the neutral case and upon charge transfer of 1.3 electrons per unit cell, respectively. (**d**) Spin-polarized electron density at the marked carbon atoms at the ribbon’s edges as a function of charge transfer.

Indeed, theoretical calculations on ribbons with different doping levels reveal important implications for their properties [31,32]. Whereas in the absence of spin polarization (3,1,6)-chGNRs display a quasi-metallic band structure (Figure 4a) [8], allowing for spin polarization results in a 16 meV more favorable ground state that includes an increased bandgap (≈238 meV) and edge states with antiferromagnetically oriented magnetization (Figure 4b). However, charging the system with 1.3 extra electrons per unit cell shifts the CNP by −0.52 eV, quenches the magnetization, and the nanoribbon recovers the quasi-metallic band structure with no spin polarization (Figure 4c).

In an attempt to quantify the necessary charge to quench the edge state magnetization, we performed additional calculations gradually modifying the GNR doping level. As depicted in Figure 4d, a charge transfer of only 0.3 electrons per unit cell is already sufficient to fully prevent any magnetism in this kind of ribbon. Although the exact value may vary for nanoribbons of different width or chirality, this is a key finding to keep in mind for the design of potential devices aiming at the exploitation of the magnetic edge states of GNRs.

In our experiment, the charge transfer throughout the whole crystal is such that it fully quenches the magnetization. However, higher work function materials may instead provide an energy level alignment that maintains the intrinsic edge state spin-polarization, and the smoothly varying work function in curved crystals as a function of the vicinal angle may help in its fine adjustment [28].

## 5. Conclusions

In conclusion, we synthesized chiral graphene nanoribbons on a curved silver crystal. Depending on the crystallographic facets displayed by the substrate steps, the ribbons grew along different orientations. At the same time, the varying step density at different vicinal angles modulated the substrate work function. As a result, angle-resolved photoemission spectroscopy at varying substrate positions allowed probing the band dispersion both along and perpendicular to the GNR axis. In addition, we could also probe the varying energy level alignment of the charge neutral point, from where the charge transfer between the GNR and substrate can be inferred. By theoretical calculations we analyzed the consequences of the latter for the ribbon’s edge state magnetization, which is fully quenched for amounts of charge transfer as low as 0.3 electrons per GNR unit cell.

## Data Availability

The data may be obtained from the corresponding authors upon reasonable request.

## Supplementary Materials

The following are available online at https://www.mdpi.com/article/10.3390/nano11123303/s1, Figure S1: ARPES raw data and substrate reference measurements. Figure S2: ARPES data across the curved silver crystal surface.
