# Supplementary material for: Band Structure and Energy Level Alignment of Chiral Graphene Nanoribbons on Silver Surfaces
Source: Nanomaterials (Basel). 2021 Dec 6;11(12):3303. doi: 10.3390/nano11123303 (PMC8705322; doi:10.3390/nano11123303)
Supplement: Supplementary file 1 [file nanomaterials-11-03303-s001.zip › nanomaterials-1483735-supplementary.pdf]

Supplementary Materials

Band Structure and Energy Level Alignment of Chiral Graphene Nanoribbons on Silver Surfaces

Martina Corso [1,2,*], Rodrigo E. Menchón [2,3], Ignacio Piquero-Zulaica [1,†], Manuel Vilas-Varela [4], J. Enrique Ortega [1,2,5], Diego Peña [3], Aran Garcia-Lekue [2,6,*] and Dimas G. de Oteyza [1,2,6,*]

[1] Centro de Física de Materiales (MPC), CSIC-UPV/EHU, 20018 San Sebastián, Spain; ge46biq@mytum.de (I.P.-Z.); enrique.ortega@ehu.es (J.E.O.)
[2] Donostia International Physics Center (DIPC), 20018 San Sebastián, Spain; menchon@dipc.org
[3] Dpto. Departamento de Polímeros y Materiales Avanzados: Física, Química y Tecnología, Universidad del País Vasco UPV/EHU, 20080 San Sebastián, Spain; diego.pena@usc.es
[4] Centro Singular de Investigación en Química Biolóxica e Materiais Moleculares (CiQUS) and Departamento de Química Orgánica, Universidade de Santiago de Compostela, 15782 Santiago de Compostela, Spain; manuel.vilas.varela@usc.es
[5] Dpto. Física Aplicada I, Universidad del País Vasco, 20018 San Sebastián, Spain
[6] Ikerbasque, Basque Foundation for Science, 48013 Bilbao, Spain
* Correspondence: martina.corso@ehu.eus (M.C.); wmbgalea@ehu.eus (A.G.-L.); d_g_oteyza@ehu.es (D.G.d.O.)
† Current address: Physics Department E20, Technical University of Munich, 85748 Garching, Germany.

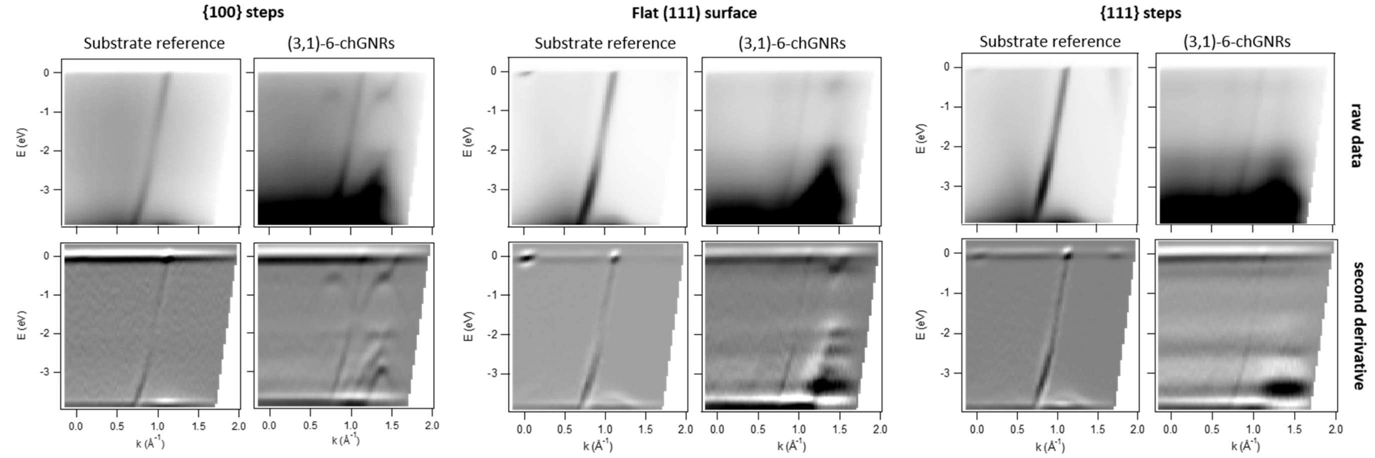

Figure S1. ARPES raw data and their second derivative for three representative regions of the GNR-covered curved Ag crystal surface (characterized by periodic {100} steps, flat (111) surface and periodic {111} steps), along with the reference map on the clean substrate. The displayed measurements are obtained at vicinal angles of approximately −10°, 0 and 10°.

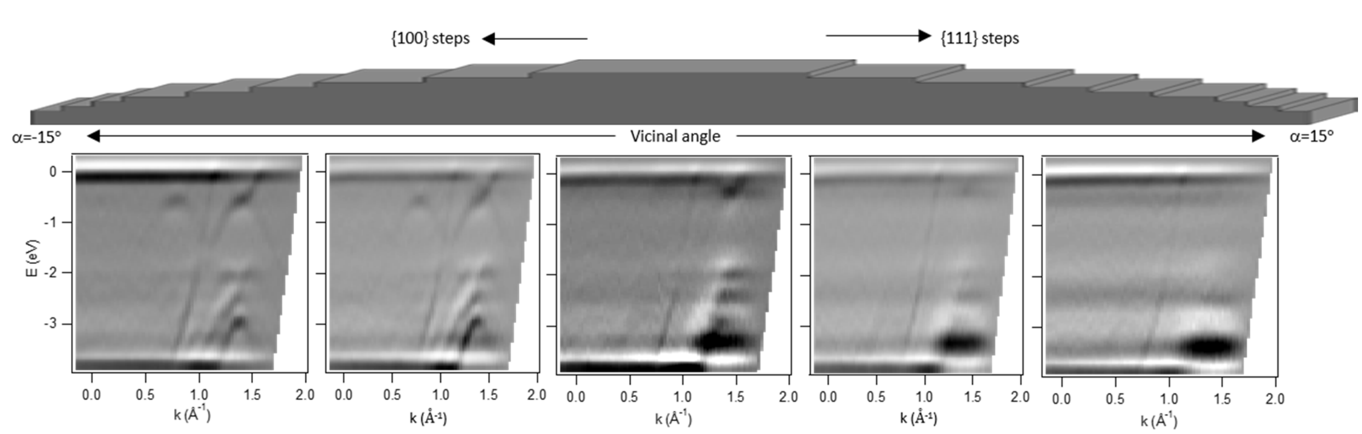

Figure S2. ARPES data obtained at five regularly spaced positions across the curved silver surface at vicinal angles of approximately −10°, −5°, 0, 5° and 10°.
